# Feasibility of using megavoltage computed tomography to reduce proton range uncertainty: A simulation study

**DOI:** 10.1002/acm2.13191

**Published:** 2021-02-20

**Authors:** Yanle Hu, Xiaoning Ding, Jiajian Shen, Martin Bues, Wei Liu, Yixiu Kang, Shuai Leng, Lifeng Yu

**Affiliations:** ^1^ Department of Radiation Oncology Mayo Clinic Phoenix AZ USA; ^2^ Department of Radiology Mayo Clinic Rochester MN USA

**Keywords:** MVCT, proton range uncertainty, stoichiometric method

## Abstract

**Purpose:**

To demonstrate that variation in chemical composition has a negligible effect on the mapping curve from relative electron density (RED) to proton stopping power ratio (SPR), and to establish the theoretical framework of using Megavoltage (MV) computed tomography (CT), instead of kilovoltage (kV) dual energy CT, to accurately estimate proton SPR.

**Methods:**

A simulation study was performed to evaluate the effect of chemical composition variation on kVCT number and proton SPR. The simulation study involved both reference and simulated human tissues. The reference human tissues, together with their physical densities and chemical compositions, came from the ICRP publication 23. The simulated human tissues were created from the reference human tissues assuming that elemental percentage weight followed a Gaussian distribution. For all tissues, kVCT number and proton SPR were obtained through (a) theoretical calculation from tissue’s physical density and chemical composition which served as the ground truth, and (b) estimation from RED using the calibration curves established from the stoichiometric method. Deviations of the estimated values from the calculated values were quantified as errors in using RED to estimate kVCT number and proton SPR.

**Results:**

Given a chemical composition variation of 5% (1σ) of the nominal percentage weights, the total estimation error of using RED to estimate kVCT number was 0.34%, 0.62%, and 0.77% and the total estimation error of using RED to estimate proton SPR was 0.30%, 0.22%, and 0.16% for fat tissues, non‐fat soft tissues and bone tissues, respectively.

**Conclusion:**

Chemical composition had a negligible effect on the method of using RED to determine proton SPR. RED itself is sufficient to accurately determine proton SPR. MVCT number maintains a superb linear relationship with RED because it is highly dominated by Compton scattering. Therefore, MVCT has great potential in reducing the proton range uncertainty.

## 1 INTRODUCTION

Proton therapy^1^, ^2^ utilizes high‐energy proton beams to deposit therapeutic level radiation dose to the tumor through direct ionization and excitation. An attractive feature of proton interaction with matter is that proton has a finite penetration depth that can be controlled through proton energy. Thus, proton therapy can completely eliminate dose deposition beyond the distal edge of the tumor (i.e., no exit dose) and achieve better dose sparing for surrounding organs‐at‐risk (OARs).^3^, ^4^, ^5^ The benefit of proton therapy, however, is partially offset by uncertainties in proton range estimation.^6^, ^7^

Although proton range may be affected by various factors such as setup and motion uncertainties, in a rigorous definition, adopted for the purpose of this work, the proton range uncertainty refers to as the uncertainty associated with the computed tomography (CT) number (in Hounsfield unit or HU) to proton stopping power ratio (SPR) calibration curve. The proton range uncertainty comes from the degeneracy effect. Materials with different chemical compositions may have the same CT number but different proton SPRs. In clinical treatment planning systems, proton SPR is determined solely from CT number using a CT number to proton SPR calibration curve. It does not require information regarding tissue chemical composition. Since the calibration curve maps one CT number to one proton SPR, it cannot handle the degeneracy effect caused by unknown chemical compositions of individual patients, resulting in the proton range uncertainty. Clinically, either an extra margin of ~3.5% of the water equivalent depth^8^, ^9^ is added to compensate the proton range uncertainty, or a similar level of the range uncertainty is included in robustness analysis.^10^, ^11^

In the current practice, the stoichiometric method^12^, ^13^ is often used to establish the CT number to proton SPR calibration curve. It uses calculated CT numbers and proton SPRs from reference human tissues, instead of measured CT numbers and proton SPRs from tissue equivalent substitutes, to minimize the bias caused by chemical composition difference between human tissues and tissue equivalent substitutes. The stoichiometric method, however, cannot address the proton range uncertainty caused by unknown tissue chemical composition of individual patients and uncertainties in mean excitation energy of water and human tissues.

To reduce proton range uncertainty, calibration methods based on dual energy CT (DECT) have been investigated.^14^, ^15^, ^16^ The most recent technical advances can be found in a review paper by Wohlfahrt et al.^17^ Dual energy CT can provide more information regarding tissue chemical composition, for example, effective atomic number (Z_eff_), and relative electron density (RED). In a theoretical investigation, Yang et al.^14^ proposed a method to derive proton SPR using Z_eff_ and RED obtained from DECT. They reported that, without image noise, kilovoltage (kV) DECT could achieve a root‐mean‐square (RMS) error of 0.26% for SPR estimation.^14^ In a subsequent simulation study, Yang et al^15^ demonstrated that the method of obtaining Z_eff_ and RED from kV‐kV DECT was very sensitive to image noise and it required use of both kV and megavoltage (MV) x rays to significantly reduce the sensitivity to image noise. But as of today kV‐MV DECT is not commercially available. Recently, Zhang et al^16^ developed a joint statistical image reconstruction (JSIR) method based on a linear basis vector model (BVM) to determine the RED and mean ionization energy from DECT images. These quantities could be subsequently used to determine proton SPR. They demonstrated in their simulation study that the JSIR‐BVM method was less sensitive to image noise. The RMS error in SPR estimation only increased from 0.2% under the idealized situation to 0.3% with image noise. The JSIR‐BVM method, however, is highly empirical and has many assumptions (e.g., selection of basis materials). Its implementation in clinical practices still requires lots of developmental efforts.

The purpose of this work was to demonstrate that the proton range uncertainty caused by unknown chemical composition of individual patients was primarily due to our inability to accurately determine kVCT number, rather than proton SPR. In a simulation study, we investigated the relationship between RED and kVCT number, as well as between RED and proton SPR, under various tissue chemical compositions. It was found out that using RED to determine proton SPR was insensitive to chemical composition variation and therefore can be used to accurately determine proton SPR. Accurate RED information can be obtained using fan‐beam or multi‐slice MVCT. Compared to kVCT, MVCT is highly dominated by Compton scattering. The photoelectric interaction in MVCT is further reduced to a negligible level, resulting in a strict linear relationship between MVCT number and RED. Based on our investigation, MVCT has great potential in reducing the proton range uncertainty.

## 2 MATERIALS AND METHODS

### 2.A Stoichiometric method

To facilitate description of evaluation, the stoichiometric method^12^ introduced by Schneider et al is reviewed briefly. The stoichiometric method includes three steps: (a) determine fitting parameters in the theoretical formula of CT number calculation using measured CT numbers and known chemical compositions of tissue equivalent substitutes, (b) calculate theoretical CT numbers and proton SPRs of the reference human tissues based on their chemical compositions provided in the International Commission on Radiological Protection (ICRP) publication 23,^18^ and (c) establish the calibration curve through segmented linear least square fitting based on calculated CT numbers and proton SPRs of the reference human tissues. The theoretical formulas to calculate the scaled CT number (abbreviated as *HU_scale_*, which is simply CT number plus 1000) and proton SPR according to tissue RED and chemical composition information are listed below.

(1) $${\mathrm{HU}}_{\mathrm{scale}}=RED∙\left(A,{\tilde{Z}}^{3.62},+,B,{\hat{Z}}^{1.86},+,C\right)$$

(2) $$SPR=RED∙\frac{\ln\left[2,m_{e},c^{2},\beta^{2},/,\left(I_{m},\left(1,‐,\beta^{2}\right)\right)\right]‐\beta^{2}}{\ln\left[2,m_{e},c^{2},\beta^{2},/,\left(I_{w},\left(1,‐,\beta^{2}\right)\right)\right]‐\beta^{2}}$$

In Eq. (1), ${\mathrm{HU}}_{\mathrm{scale}}=\mathrm{HU}+1000$, $\tilde{Z}={\left[\sum ,\lambda_{i},Z_{i}^{3.62}\right]}^{1/3.62}$, $\hat{Z}={\left[\sum ,\lambda_{i},Z_{i}^{1.86}\right]}^{1/1.86}$, and $\lambda_{i}=N_{g}^{i}/N_{g}$. Here, $N_{g}$ and $N_{g}^{i}$ are the total number of electrons per unit mass and the number of electron per unit mass contributed by the i^th^ element, respectively. Given $Z_{i}$, $A_{i}$, and $\omega_{i}$ are the atomic number, atomic weight and weight proportion for the i^th^ element and $N_{A}$ is the Avogadro’s number, then $N_{g}=\sum N_{g}^{i}=N_{A}\sum \left(\omega_{i},Z_{i},/,A_{i}\right)$. A, B, and C are fitting parameters which are machine and energy dependent. The three terms in Eq. (1) correspond to photoelectric interaction, coherent scattering and Compton scattering, respectively, because all existing CT simulators operate in the kV energy range (70–140 kVp). In Eq. (2), $m_{e}$ is the rest mass of electron, c is the speed of light, β is a dimensionless quantity defined as the ratio of proton speed to light speed (v/c). $I_{m}$ and $I_{w}$ are the mean ionization energy of the tissue and water, respectively. $I_{m}$ is calculated from the ionization energy $I_{i}$ for each element using Eq. (3). Ionization energies of individual elements and water can be found in Geant4 manual (“Book for Application Developers”, Release 10.7, Geant4 Collaboration, https://geant4.web.cern.ch/). In both Eqs. (1) and (2), RED is calculated using Eq. (4) where ρ, $N_{g}$, $\rho_{w}$, and $N_{w}$ are tissue density, tissue total number of electrons per unit mass, water density and water total number of electrons per unit mass, respectively.

(3) $$\ln I_{m}=\left(\sum ,\frac{\omega_{i}Z_{i}}{A_{i}},\ln,I_{i}\right)/\left(\sum ,\frac{\omega_{i}Z_{i}}{A_{i}}\right)$$

(4) $$RED=\frac{\rho N_{g}}{\rho_{w}N_{w}}$$

The final output of the stoichiometric method is a calibration curve that can be used in treatment planning systems to convert CT number to proton SPR. Thus, it is also called the calibration curve method. The calibration curve method is what we use in the clinical practice. It does not require tissue chemical composition to determine proton SPR.

### 2.B Determining fitting parameters for kVCT number calculation

A CIRS 062M electron density phantom (CIRS, Norfolk, Virginia, USA) was used to determine the fitting parameters A, B, and C in Eq. (1). The phantom has 14 tissue equivalent substitutes, including Lung Inhale, Lung Exhale, Adipose, Breast 50/50, Plastic Water, True Water, Muscle, Liver, Bone 200 mg/cc, Bone 800 mg/cc, Bone 1000 mg/cc, Bone 1250 mg/cc, Bone 1500 mg/cc, and Bone 1750 mg/cc. Physical densities and chemical compositions of these tissue equivalent substitutes were obtained from the vendor.

A Siemens CT simulator (SOMATOM Definition AS‐20, Siemens Healthineers, Erlangen, Germany) was used to measure kVCT numbers of tissue equivalent substitutes. The phantom was setup on the CT couch using the abdomen configuration and sent to the center of the CT simulator. CT scans were performed for all 14 tissue equivalent substitutes, one at a time, using helical CT acquisition with 120 kVp, 300 mAs, pitch of 0.8, slice thickness of 5 mm and J30 Safire filter. For each tissue equivalent substitute, 5 CT scans were performed with the tissue equivalent substitute placed at the center, 3 o’clock, 6 o’clock, 9 o’clock, and 12 o’clock. The off‐center locations were 115, 110, 115, and 115 mm from the phantom center, and 50, 20, 50, and 25 mm from the phantom edge for 3, 6, 9, and 12 o’clock locations, respectively. The average CT numbers across five locations were used to determine the fitting parameters in the MATLAB (Version R2018a, The MathWorks, Inc., Natick, MA, USA) through least square fitting.

### 2.C Reference and simulated human tissues

The reference human tissues used in this study were from the ICRP publication 23.^18^ Physical densities and chemical compositions of the reference human tissues were listed in Table 1. Two tissues, cell nucleus and breast, were excluded from the study. Cell nucleus was excluded because it never exists by itself in human body. Breast was excluded because the reported chemical composition was for the entire breast which was a mixture of fat and glandular tissues. However, on CT images at the voxel level, fat and glandular tissues are clearly separated. Voxels in breast belong to either fat or glandular tissues except for a few voxels near the tissue boundary (i.e., the partial volume effect). Based on similarity in chemical composition, tissues were grouped into fat tissues, non‐fat soft tissues and bone tissues, as shown in Table 1.

**TABLE 1:** Physical densities (g/cm^3^) and chemical compositions (%) of the reference human tissues.

	ρ	H	C	N	O	Ca	P	Na	Mg	S	Cl	K	Fe	I
Fat tissues														
Adipose	0.95	11.4	59.8	0.7	27.8			0.1		0.1	0.1			
Yellow marrow	0.98	11.5	64.4	0.7	23.1			0.1		0.1	0.1			
Red marrow	1.03	10.5	41.4	3.4	43.9		0.1			0.2	0.2	0.2	0.1	
Non‐fat soft tissues														
Lung (inflated)	0.26	10.3	10.5	3.1	74.9		0.2	0.2		0.3	0.3	0.2		
GI tract	1.03	10.6	11.5	2.2	75.1		0.1	0.1		0.1	0.2	0.1		
Lymph	1.03	10.8	4.1	1.1	83.2			0.3		0.1	0.4			
Brain	1.04	10.7	14.5	2.2	71.2		0.4	0.2		0.2	0.3	0.3		
Pancreas	1.04	10.6	16.9	2.2	69.4		0.2	0.2		0.1	0.2	0.2		
Testis	1.04	10.6	9.9	2	76.6		0.1	0.2		0.2	0.2	0.2		
Kidney	1.05	10.3	13.2	3	72.4	0.1	0.2	0.2		0.2	0.2	0.2		
Lung (deflated)	1.05	10.3	10.5	3.1	74.9		0.2	0.2		0.3	0.3	0.2		
Muscle	1.05	10.2	14.3	3.4	71		0.2	0.1		0.3	0.1	0.4		
Ovary	1.05	10.5	9.3	2.4	76.8		0.2	0.2		0.2	0.2	0.2		
Thyroid	1.05	10.4	11.9	2.4	74.5		0.1	0.2		0.1	0.2	0.1		0.1
Blood	1.06	10.2	11	3.3	74.5		0.1	0.1		0.2	0.3	0.2	0.1	
Heart	1.06	10.3	12.1	3.2	73.4		0.1	0.1		0.2	0.3	0.2	0.1	
Liver	1.06	10.2	13.9	3	71.6		0.3	0.2		0.3	0.2	0.3		
Spleen	1.06	10.3	11.3	3.2	74.1		0.3	0.1		0.2	0.2	0.3		
Eye lens	1.07	9.6	19.5	5.7	64.6		0.1	0.1		0.3	0.1			
Skin	1.09	10	20.4	4.2	64.5		0.1	0.2		0.2	0.3	0.1		
Cartilage	1.1	9.6	9.9	2.2	74.4		2.2	0.5		0.9	0.3			
Bone tissues														
Skeleton‐ spongiosa	1.18	8.5	40.4	2.8	36.7	7.4	3.4	0.1	0.1	0.2	0.2	0.1	0.1	
Skeleton‐ sacrum	1.29	7.4	30.2	3.7	43.8	9.8	4.5		0.1	0.2	0.1	0.1	0.1	
Skeleton‐ femur	1.33	7	34.5	2.8	36.8	12.9	5.5	0.1	0.1	0.2	0.1			
Skeleton‐ vertebra (L3)	1.33	7	28.7	3.8	43.7	11.1	5.1		0.1	0.2	0.1	0.1	0.1	
Skeleton‐ ribs (2‐6)	1.41	6.4	26.3	3.9	43.6	13.1	6	0.1	0.1	0.3	0.1	0.1		
Skeleton‐ vertebra (C4)	1.42	6.3	26.1	3.9	43.5	13.3	6.1	0.1	0.1	0.3	0.1	0.1	0.1	
Skeleton‐ humerus	1.46	6	31.4	3.1	36.9	15.2	7	0.1	0.1	0.2				
Skeleton‐ ribs (10)	1.52	5.6	23.5	4	43.4	15.6	7.2	0.1	0.1	0.3	0.1	0.1		
Skeleton‐ cranium	1.61	5	21.2	4	43.5	17.6	8.1	0.1	0.2	0.3				
Skeleton‐ mandible	1.68	4.6	19.9	4.1	43.5	18.7	8.6	0.1	0.2	0.3				
Skeleton‐ cortical bone	1.92	3.4	15.5	4.2	43.5	22.5	10.3	0.1	0.2	0.3				

The simulated human tissues were generated from the reference human tissues. Assuming that human tissue elemental percentage weights follow the Gaussian distribution, the simulated human tissues were created by sampling the Gaussian distribution based on the mean (µ) and standard deviation (σ) of the percentage weight for each element. From each reference human tissue, 100 simulated human tissues were generated. In the process of generating the simulated human tissues, we used the elemental percentage weights of the reference human tissue as the mean and 5% of the elemental percentage weights of the reference human tissue as the standard deviation.

Creation of the simulated human tissues was performed using the MATLAB software (Version R2018a, The MathWorks, Inc., Natick, MA, USA). It involved multiple steps. First, a reference tissue was selected. Second, 100 random samples were generated for each element based on the element’s mean percentage weight and standard deviation. Third, in a sequential order, 100 samples from all elements were put together to form 100 combinations of the chemical composition. Fourth, for each combination, the sum of percentage weights from all elements was normalized to 100% to mimic realistic scenarios. At this point, 100 simulated human tissues were created from that specific reference human tissue. Fifth, we repeated step 1–4 for all reference human tissues. In total, 3200 simulated human tissues were created from 32 reference human tissues.

### 2.D Quantification of uncertainties

For all reference and simulated human tissues, kVCT number and proton SPR were calculated using two methods. The first method used Eqs. (1) and (2) to calculate kVCT number and proton SPR according to the exact chemical compositions of the reference and simulated human tissues. These values served as the ground truth for error estimation. The second method mimicked the clinical scenario in which tissue chemical composition was not available and kVCT number and proton SPR were obtained from RED using the calibration curve method. To evaluate individual effects of chemical composition on kVCT number and proton SPR, two calibration curves were established using the stoichiometric method from RED to kVCT number and from RED to proton SPR, instead of one calibration curve directly from kVCT number to proton SPR as in previous studies. To improve accuracy, all calibration curves were established through segmented linear least square fitting based on individual tissue categories to avoid large tissue chemical composition variation.

KVCT numbers and proton SPRs estimated using the calibration curve method were compared to those calculated directly from tissue chemical compositions using Eqs. (1) and (2). The deviations were then normalized to the ground truth values of kVCT numbers and proton SPRs obtained using the first method to get the percentage deviations. Uncertainties in kVCT number and proton SPR estimation using the clinically implemented calibration curve method were quantified through the standard deviation of the percentage deviations. The uncertainty was divided into two components: systematic uncertainty and statistical uncertainty. The systematic uncertainty was quantified using the reference human tissues whereas the statistical uncertainty was quantified using the simulated human tissues. For statistical uncertainty quantification, the systematic effect of chemical composition variation was separated and removed by subtracting the deviation caused by the reference human tissue from which the simulated human tissues was generated. Based on the systematic and statistical uncertainties, the total uncertainty was obtained through the root sum squared.

### 2.E Comparison of kVCT and MVCT using Monte Carlo simulation

To demonstrate sensitivities of kVCT and MVCT numbers to chemical composition variation, we performed Monte Carlo simulation for both kV and MV beams using a single energy. Since accurate energy spectrum information was not available to us, we did a coarse estimation and used 60 keV for the 120 kVp beam because this was close to the characteristic x‐ray energies of tungsten and 0.8 MeV for the 2.5 MV imaging beam because it was close to the average energy. In the Monte Carlo simulation, we focused primarily on the mapping from RED to kVCT and MVCT numbers (equivalent to the mapping from kVCT and MVCT numbers to RED for the purpose of evaluating the effect of chemical composition variation). This was because the mapping from RED to proton SPR was the same no matter kV or MV beams were used.

To calculate CT numbers for kV and MV beams, simulation code based on Geant4 (Geant4 Collaboration, https://geant4.web.cern.ch/) was developed to obtain phase‐space files behind the phantom, for an infinitively small photon beamlet^19^, ^20^ at 60 keV or 0.8 MeV. For each run, the phantom was filled with a single type of material, either water or one of the 32 reference human tissues as defined in Table 1. The physics model used in the Monte Carlo simulation was the built‐in “QGSP_BIC_EMY” model. 12 million photon particles were used in the simulation. The phase‐space files contained particle information such as particle type, position, momentum and energy, that went through a plane perpendicular to the beamlet at 1 meter downstream from the phantom. The number of un‐scattered photons, which went through the phantom without any interaction with the phantom material, were obtained from the phase‐space file. The ratio of the un‐scattered photons over the incident photons was used to calculate the photon attenuation coefficient. The scaled CT numbers (CT number plus 1000) for both kV and MV beams were calculated from the attenuation coefficients using Eq. (5). For comparison, the scaled kV and MV CT numbers were plotted against RED in the same figure.

(5) $${\mathrm{HU}}_{\mathrm{scale}}=1000+1000\times \left(\mu ,‐,\mu_{\mathrm{water}}\right)/\mu_{\mathrm{water}}$$

## 3 RESULTS

**FIG. 1:**
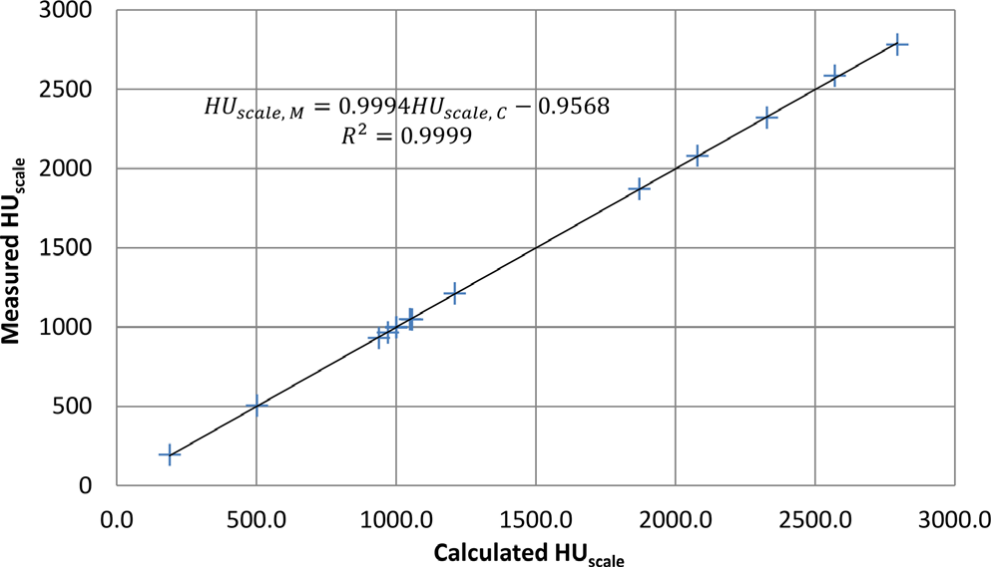
Plot of the measured ${\mathrm{HU}}_{\mathrm{scale}}$ vs the calculated ${\mathrm{HU}}_{\mathrm{scale}}$, demonstrating excellent fitting quality.

The average kVCT HU_scale_ for Lung Inhale, Lung Exhale, Adipose, Breast 50/50, Plastic Water, True Water, Muscle, Liver, Bone 200 mg/cc, Bone 800 mg/cc, Bone 1000 mg/cc, Bone 1250 mg/cc, Bone 1500 mg/cc, and Bone 1750 mg/cc was 193.7, 505.3, 930.7, 966.1, 998.3, 999.8, 1048.9, 1047.5, 1212.2, 1871.7, 2079.9, 2321.0, 2583.4, and 2787.0, respectively. Using the measured average HU_scale_ and the vendor supplied chemical composition of tissue equivalent substitutes, it was determined that the fitting parameters in Eq. (1) were A = 1.995 × 10^−2^, B = 1.899 × 10^−1^ and C = 9.640 × 10^2^. Once the fitting parameters were determined, HU_scale_ of tissue equivalent substitutes could also be calculated based on Eq. (1). Figure 1 plotted the measured HU_scale_ against the calculated HU_scale_ for all tissue equivalent substitutes, demonstrating excellent fitting quality (R^2^ = 0.9999). The percentage deviations were all <1% except for the Lung Inhale substitute (2%) due to its very low density.

**FIG. 2:**
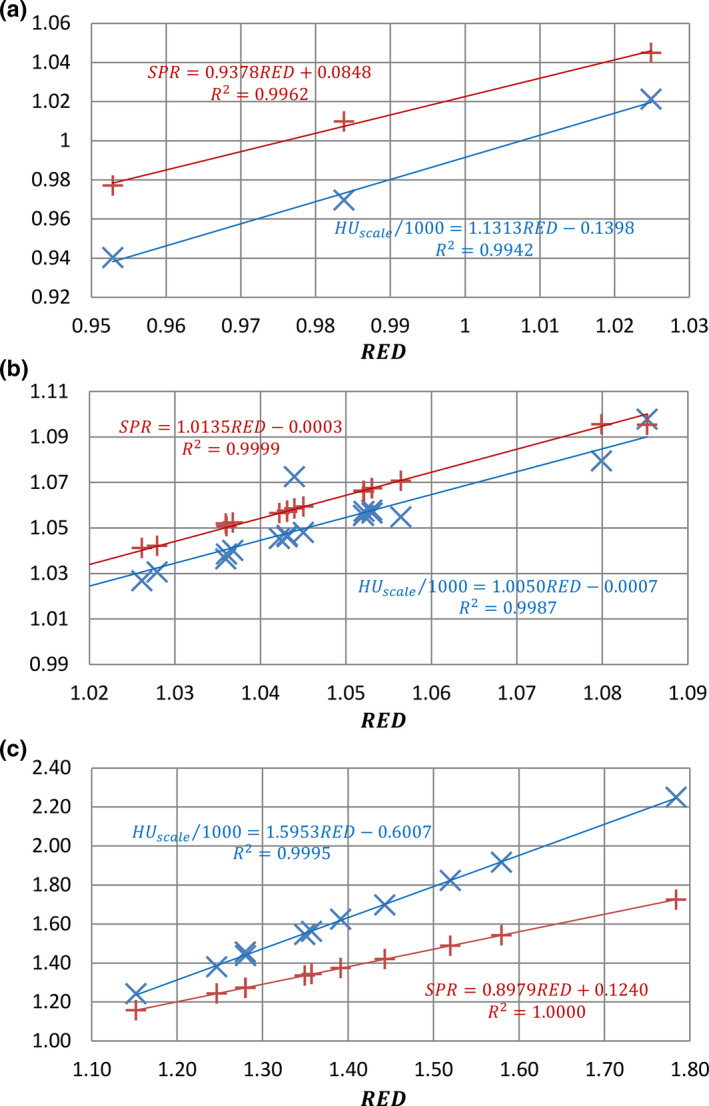
${\mathrm{HU}}_{\mathrm{scale}}/1000$ and SPR vs RED for (a) fat tissues, (b) non‐fat soft tissues, and (c) bone tissues. In general, SPR deviates less from the fitting curves than ${\mathrm{HU}}_{\mathrm{scale}}/1000$.

Using the first method described in the section of “Quantification of Uncertainties,” HU_scale_ and proton SPR were calculated for all reference and simulated human tissues using Eqs. (1) and (2), as well as their known chemical compositions. Using the calibration curve method (or the second method), HU_scale_ and proton SPR were calculated directly from RED for all reference and simulated human tissues without using tissue chemical composition information. For the second method, the calibration curves were first established from RED to kVCT number and from RED to proton SPR using the reference human tissues. Figures 2(a), 2(b), and 2(c) corresponded to fat tissues, non‐fat soft tissues and bone tissues, respectively. ${\mathrm{HU}}_{\mathrm{scale}}$ was normalized to water (i.e., ${\mathrm{HU}}_{\mathrm{scale}}/1000$) to facilitate display and comparison. Figure 2 shows that SPR deviates less from the fitting curves than ${\mathrm{HU}}_{\mathrm{scale}}/1000$, as demonstrated by higher R^2^ in the figure.

**FIG. 3:**
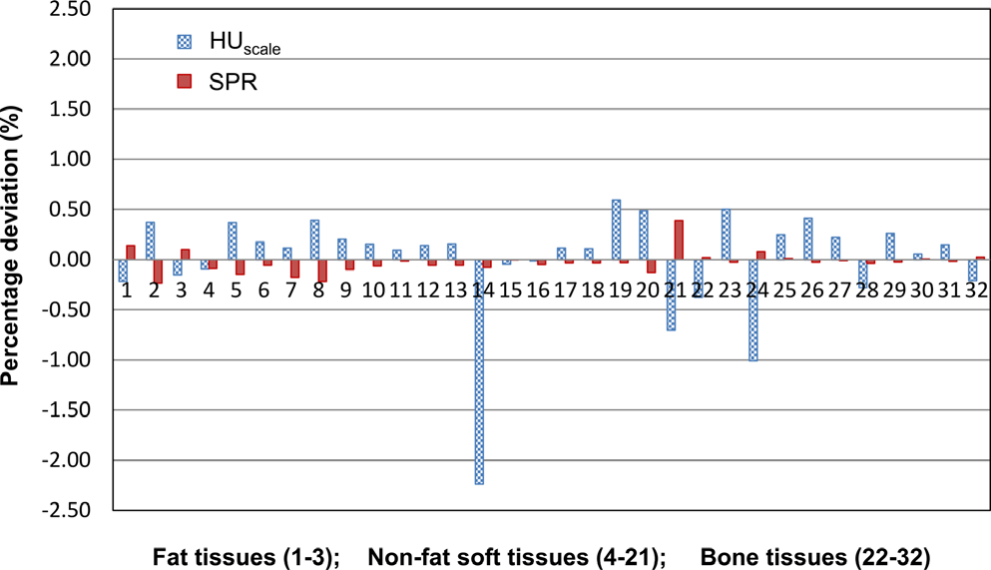
Percentage deviations in kVCT ${\mathrm{HU}}_{\mathrm{scale}}$ and proton SPRbetween the calibration curve method and the method based on Eq. (1) and (2) (or the first method in the section of "Quantification of uncertainties") for the reference human tissues.

**FIG. 4:**
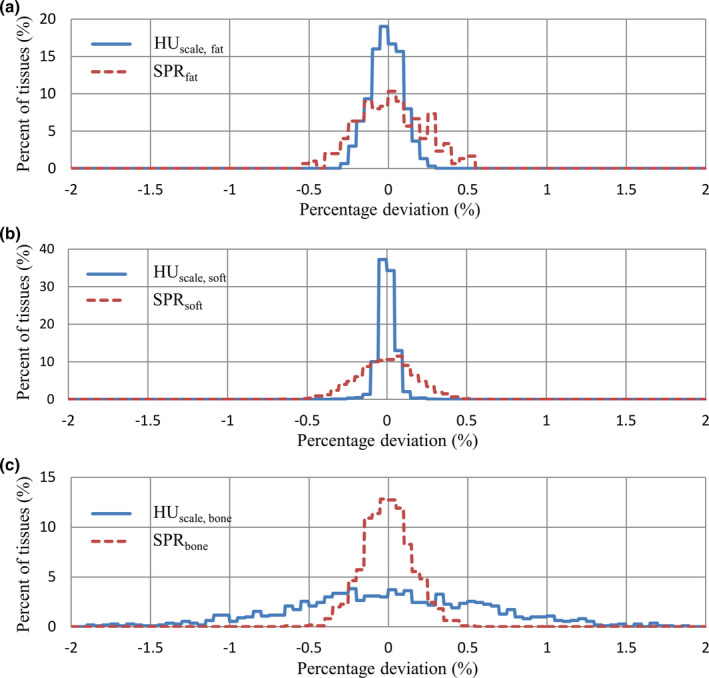
Histograms of percentage deviations, excluding the systematic effect, for the simulated (a) fat tissues, (b) non‐fat soft tissues and (c) bone tissues.

Figure 3 shows percentage deviations in kVCT HU_scale_ and proton SPR between the two methods described in the previous section for the reference human tissues. These were used to obtain the systematic uncertainty (1δ). Figure 4 plots histograms of percentage deviations, excluding the systematic effect, for the simulated human tissues. These were used to obtain the statistical uncertainty (1σ). Table 2 summarizes the quantitative results for systematic, statistical and total uncertainties, as well as maximum absolute percentage deviation, in kVCT HU_scale_ and proton SPR estimation using the clinically implemented calibration curve method. It demonstrates that the systematic uncertainty in kVCT HU_scale_ is much higher than that in proton SPR among all tissue categories. The statistical uncertainty in kVCT HU_scale_, however, is low for fat and non‐fat soft tissues but high for bone tissues, compared to that in proton SPR. It is consistent with assumption that the kVCT HU_scale_ is more sensitive to high Z elements (e.g., Ca) which have a higher proportion in bone tissues. Combining the systematic and statistical uncertainties, the total uncertainty among all tissue categories is 0.65% for kVCT HU_scale_ and 0.21% for proton SPR.

**TABLE 2:** Systematic, statistical, and total uncertainties, as well as maximum absolute percentage deviation, in kVCT HU_scale_ and proton SPR estimation using the calibration curve method.

	Systematic		Statistical		Total	
	HU_scale_	SPR	HU_scale_	SPR	HU_scale_	SPR
Uncertainty						
Fat tissues	0.32%	0.21%	0.11%	0.21%	0.34%	0.30%
Non‐fat soft tissues	0.62%	0.12%	0.06%	0.19%	0.62%	0.22%
Bone tissues	0.44%	0.03%	0.63%	0.16%	0.77%	0.16%
All tissues	0.53%	0.11%	0.38%	0.18%	0.65%	0.21%
Maximum absolute percentage deviation						
Fat tissues	0.37%	0.24%	0.32%	0.59%	—	—
Non‐fat soft tissues	2.24%	0.39%	0.38%	0.72%	—	—
Bone tissues	1.01%	0.08%	2.62%	0.51%	—	—
All tissues	2.24%	0.39%	2.62%	0.72%	—	—

**FIG. 5:**
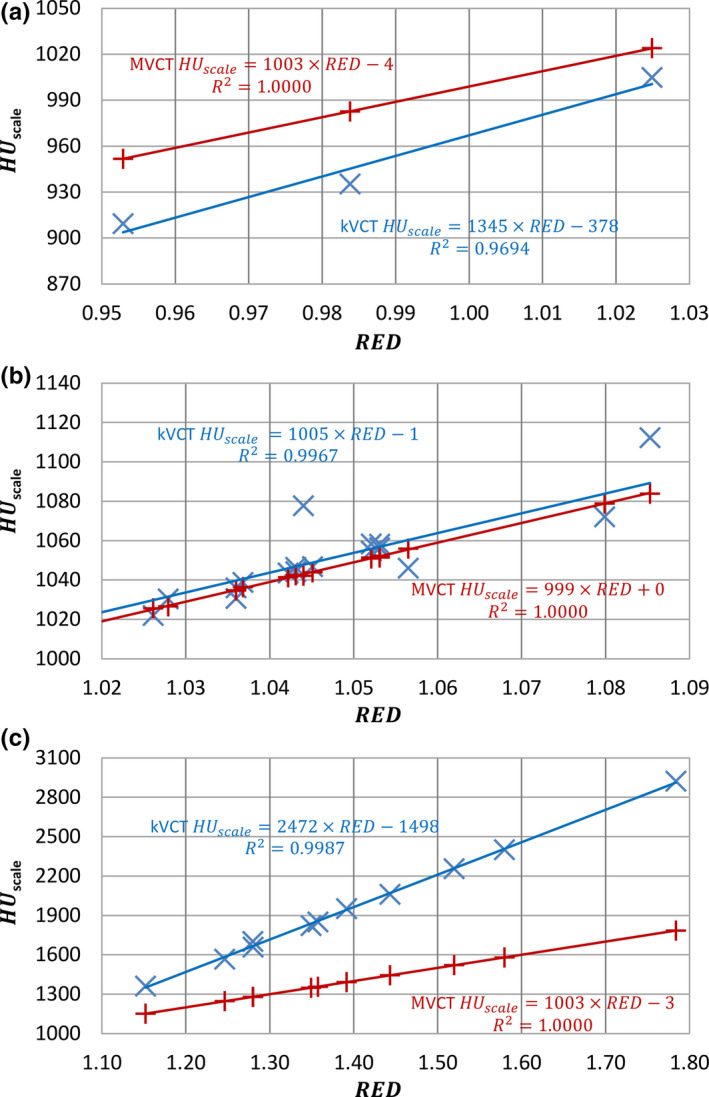
kVCT and MVCT ${\mathrm{HU}}_{\mathrm{scale}}$ vs RED for (a) fat tissues, (b) non‐fat soft tissues, and (c) bone tissues.

Figure 5 plotted the mapping curves from RED to kVCT and MVCT numbers for fat tissues, non‐fat soft tissues and bone tissues. It can be seen the mapping curves from RED to MVCT number have a better linear relationship (R^2^ = 1.0000, 1.0000, and 1.0000 for fat, non‐fat soft tissue, and bone, respectively) compared to the mapping curves from the RED to kVCT number (R^2^ = 0.9694, 0.9967, and 0.9987 for fat, non‐fat soft tissue, and bone, respectively), indicating MVCT number is less sensitive to chemical composition variation compared to kVCT number. Therefore, MVCT has the potential to reduce proton range uncertainty.

## 4 DISCUSSION

In this work, we demonstrated that when kVCT was used to estimate proton SPR, the primary source of range uncertainty came from the inability to accurately determine kVCT number, rather than proton SPR. In fact, proton SPR was quite insensitive to chemical composition variation. Based on our investigation, using RED to determine SPR could achieve a systematic uncertainty of 0.11% among all reference human tissues. As a comparison, the systematic uncertainty of using kVCT to determine proton SPR was 0.89%.^14^ Using kV DECT to determine proton SPR could potentially reduce the proton range uncertainty.^17^ Under the ideal (or noiseless) condition, the reported systematic uncertainty of using kV DECT to determine proton SPR was 0.26% in Yang et al’s study^14^ and 0.16% in Zhang et al’s study.^16^ For the statistical uncertainty, our study showed that using RED to determine proton SPR could achieve a statistical uncertainty of 0.18% among all simulated human tissues, which was lower than the reported statistical uncertainty that can be achieved by kVCT (0.18%, 1.2%, and 1.6% for lung, soft, and bone tissues, respectively).^9^

In the process of obtaining the statistical uncertainty, 5% of elemental percentage weight was used as the standard deviation to create the simulated human tissues. It was a rough estimation based on a literature review conducted by Yang et al.^9^ In their study, they estimated the standard deviation relative to element’s nominal percentage weight was about 4.8% for H and 4.4% for Ca. To simplify the simulation process, we used 5% for all elements. Due to insufficient data available to us, we could not verify how close our assumption matched the ground truth. Thus, it represents a limitation of our study. Another limitation is that the current study only considered the uncertainty caused by chemical composition variation. It did not consider the uncertainty in the calibration curve caused by uncertainties in mean ionization energies of water and human tissues. In a previous study, Andreo^21^ demonstrated that different water mean ionization energy reported in the literature (67, 75, or 80 eV) might contribute to an absolute Bragg peak depth variation of 5–6 mm for protons and heavier charged particles. However, in our study, we focused on proton stopping power ratio relative to water. Yang et al^9^ demonstrated that a 10% variation in elemental ionization energy only resulted in 0.17%, 0.23%, and 0.65% uncertainty in proton SPR for lung, soft, and bone tissues, respectively.

In Figures 2 and 3, there is an obvious outlier tissue. The outlier tissue is thyroid. Thyroid has 0.1% of iodine (Z = 53). The low concentration of iodine can change the effective atomic number ($\tilde{Z}$) of thyroid. Even though the change is small, the high sensitivity of kVCT number to $\tilde{Z}$ results in a noticeable deviation from the fitting line from RED to kVCT number. As a result, thyroid becomes an outlier. On the other hand, the logarithm operation in proton SPR calculation makes it insensitive to chemical composition variation. Thus, thyroid’s proton SPR remains close to the fitting line from RED to proton SPR. This is a good example that uncertainty due to tissue chemical composition is more on the kVCT number side, rather than on the proton SPR side.

Accurate determination of RED is feasible using fan‐beam or multislice MVCT. In fact, even in the energy range of kVCT, Compton scattering is the dominant interaction. Thus, kVCT number maintains a reasonable linear relationship with RED when tissues are grouped into three categories (fat, non‐fat soft tissue, and bone) based on the similarity of their chemical compositions. The systematic uncertainty of using RED to predict kVCT number is 0.53%. As we move to MVCT, the percentage of low energy photons drops. It further reduces the contributions of photoelectric interaction and coherent scattering to a negligible level. As a result, Eq. (1) reduces to ${\mathrm{HU}}_{\mathrm{scale}}=RED∙C$ where C is a constant. Thus, MVCT number maintains a superb linear relationship with RED and can be used to accurately determine RED. Given the fact that RED maintains a superb linear relationship with proton SPR, using MVCT number to determine proton SPR has a great potential to reduce proton range uncertainty. One thing to note is that cone‐beam MVCT can also provide MVCT numbers, but it may not be suitable for accurate RED determination. This is because there are larger number of scatter photons in cone‐beam MVCT which not only reduce image contrast but also introduce systematic deviations in MVCT numbers from their expected values. Therefore, use of cone‐beam MVCT number to determine RED may not have sufficient accuracy.

Although the superb linear relationship was demonstrated qualitatively in previous work between RED and proton SPR,^22^ as well as between MVCT number and proton SPR,^23^ none of these studies provided quantitative results regarding the uncertainty of using RED or MVCT number to predict proton SPR, especially under the condition of chemical composition variation. In this work, we quantified the systematic, statistical and total uncertainties of using RED to predict kVCT number and proton SPR. The results clearly demonstrated that most uncertainties were due to our inability to accurately determine kVCT number, rather than proton SPR (0.65% vs 0.21% for the total uncertainty). If we use MVCT, instead of kVCT, to predict proton SPR, we can reduce the sensitivity of the calibration curve to chemical composition variation and improve the accuracy of proton range estimation.

Currently, well calibrated fan‐beam MVCT is not commercially available. However, with extra developmental work, MVCT may become available using existing radiotherapy hardware. One potential solution is Tomotherapy. Tomotherapy offers fan‐beam MVCT. But its focus is more on patient alignment rather than acquiring simulation images. With appropriate calibration, Tomotherapy does have the potential to provide MVCT that is good for the simulation purpose. Another possible solution is to utilize radiotherapy systems equipped with the EPID panel (e.g., Varian TrueBeam). Using the MLC system, it is possible to collimate the MV beam from cone‐beam geometry to fan‐beam geometry required by fan‐beam MVCT. This solution does require substantial developmental work on image acquisition, reconstruction and calibration. For radiotherapy system with MV CBCT capability (e.g., Varian Halcyon), it is also possible to collimate the MV beam from cone‐beam geometry to fan‐beam geometry to achieve fan‐beam MVCT. This solution is slightly simpler because we may be able to directly utilize the vendor supplied reconstruction algorithms. But additional efforts on image acquisition and calibration are still necessary.

Given the fact that well calibrated fan‐beam MVCT is not commercially available, we used Monte Carlo simulation to compare sensitivities of kVCT and MVCT numbers to chemical composition variation. A limitation of our Monte Carlo simulation is use of single energy in the simulation because the energy spectrums of the kV and MV beams were not available to us. Even though the Monte Carlo simulation was quantitative, it may not include all effects. This is because that the degeneracy effect is caused by the photoelectric interaction which is mostly sensitive to low‐energy photon (~30 keV). Thus, the percentage of low energy photon also contributes to the overall effect of the photoelectric interaction (or sensitivity to the degeneracy effect). From kV CT to MV CT, the percentage of low energy photons drops. Thus, the contribution from the photoelectric interaction drops, making MVCT less sensitive to the degeneracy effect. If we use monochroic MV beam, the drop in the percentage of low‐energy photons from kVCT to MVCT is not considered.

Developing fan‐beam MVCT using existing radiotherapy hardware faces several challenges. One of these challenges is related to the slow image acquisition. For the potential solution that utilizes the MV beam, MLC and EPID, the speed of image acquisition is limited by the slow gantry rotation and the fact that the gantry can only rotate slightly more than 360°, instead of continuous rotation like regular CT simulators. Slow image acquisition increases the simulation time and also makes MVCT more susceptible to blurring caused by motion during image acquisition. Another technical challenge is related with imaging dose. Compared to the kV imaging system, the MV detectors have lower detection efficiency and thus require a higher dose to achieve the same noise level. To minimize image acquisition time, we can collimate the MV beam so that it is in between fan‐beam and cone‐beam geometries (similar to multislice CT) and also focus only on the area around the treatment target. To minimize imaging dose, we can explore iterative reconstruction algorithms and advanced MV detector panels. These are all interesting topics and will be investigated in future studies.

Due to the long image acquisition time and added imaging dose, Fan‐beam MVCT may not be suitable for all disease sites. Use of fan‐beam MVCT requires clinical evaluation of risks and benefits. For prostate cancer, the added dose calculation accuracy may not be clinically significant. Thus, fan‐beam MVCT may not be necessary. But for Head and Neck cases or base of skull cases, due to close proximity of the treatment target to organs at risk (brain stem, cord, etc), accurate dose calculation is crucial and use of fan‐beam MVCT to optimize treatment plan and calculate dose may become justifiable.

Compared to simultaneous DECT, use of MVCT together with traditional kV CT simulation requires additional image registration and may introduce an extra uncertainty. But the registration error is expected to be small for cases that benefit most from MVCT, for example, Head and Neck, base of skull etc. In addition, we feel this uncertainty should be in the category of setup uncertainty, instead of the uncertainty caused by chemical composition variation. Although DECT does not require image registration, patient position may still vary between simulation and treatment, causing deviation in planned and delivered doses. MVCT does require image registration, but it is reasonable to assume its position is closer to the treatment position and therefore may help minimize deviation between planned and delivered doses. In both cases, this can be sufficiently covered by the setup margin. Using MVCT to determine proton SPR has some additional advantages compared to kVCT. First, it eliminates additional image processing that is required to obtain physical quantities from DECT images. Thus, it is more robust to image noise. Second, MVCT is less sensitive to the beam hardening effect due to its higher penetration power. Third, it has significantly less imaging artifacts from metal implants.

The main purpose of the manuscript was to demonstrate the potential of MVCT in reducing the proton range uncertainty. Other promising methods to reduce the proton range uncertainty include DECT and proton CT. In fact, DECT and proton CT have been investigated extensively, but MVCT, as an alternative method, has not received enough investigation. There are many challenges, for example, image acquisition time, imaging dose, etc., that need to be sufficiently addressed before MVCT can be evaluated for clinical use and compared with other promising methods like DECT and proton CT. We hope that by demonstrating the potential of MVCT, we can stimulate more interests in the development of fan‐beam MVCT as it may provide a practical solution to reduce the proton range uncertainty in future.

## 5 CONCLUSION

We demonstrated that chemical composition variation had a negligible effect on the method of using RED to determine proton SPR. Therefore, RED itself may be sufficient to accurately determine proton SPR. MVCT number maintains a superb linear relationship with RED because it is highly dominated by Compton scattering. Thus, MVCT has great potential in reducing the proton range uncertainty.

## AUTHOR CONTRIBUTION STATEMENT

YH, XD, JS, MB, WL, YK, SL, and LY contributed to the study design, interpretation of results, and preparation of the manuscript. YH and XD contributed to the writing of simulation codes and data analysis.

## CONFLICT OF INTEREST

There is no conflict of interest.
